# Paving the Way for a Green Transition in the Design of Sensors and Biosensors for the Detection of Volatile Organic Compounds (VOCs)

**DOI:** 10.3390/bios12020051

**Published:** 2022-01-19

**Authors:** Camilla Maria Cova, Esther Rincón, Eduardo Espinosa, Luis Serrano, Alessio Zuliani

**Affiliations:** 1 Department of Chemistry, University of Florence and CSGI, Via della Lastruccia 3, 50019 Sesto Fiorentino, FI, Italy; cova@csgi.unifi.it; 2 BioPren Group, Inorganic Chemistry and Chemical Engineering Department, Faculty of Sciences, University of Cordoba, 14014 Cordoba, Spain; b32rirue@uco.es (E.R.); eduardo.espinosa@uco.es (E.E.); iq3secal@uco.es (L.S.)

**Keywords:** biosensors, VOCs, environmental, packaging, diagnostic, pollution

## Abstract

The efficient and selective detection of volatile organic compounds (VOCs) provides key information for various purposes ranging from the toxicological analysis of indoor/outdoor environments to the diagnosis of diseases or to the investigation of biological processes. In the last decade, different sensors and biosensors providing reliable, rapid, and economic responses in the detection of VOCs have been successfully conceived and applied in numerous practical cases; however, the global necessity of a sustainable development, has driven the design of devices for the detection of VOCs to greener methods. In this review, the most recent and innovative VOC sensors and biosensors with sustainable features are presented. The sensors are grouped into three of the main industrial sectors of daily life, including environmental analysis, highly important for toxicity issues, food packaging tools, especially aimed at avoiding the spoilage of meat and fish, and the diagnosis of diseases, crucial for the early detection of relevant pathological conditions such as cancer and diabetes. The research outcomes presented in the review underly the necessity of preparing sensors with higher efficiency, lower detection limits, improved selectivity, and enhanced sustainable characteristics to fully address the sustainable manufacturing of VOC sensors and biosensors.

## 1. Introduction

The United States Environmental Agency (EPA) and the European Environmental Agency (EEA) define as a volatile organic compound (VOC) any organic substance that under normal conditions is gaseous or can vaporize in the atmosphere [1,2]. Although this general description helps in easily recognizing a volatile organic compound, it is too rough and is not unequivocal in identifying VOCs. Therefore, different national and international regulations have proposed more standardized definitions according to selected physico-chemical properties of the considered chemicals. Among all, the EU Council Directive 1999/13/EC (and successive amendments and corrections) indicates as a VOC “any organic compound having at 20 °C a vapor pressure of 0.01 kPa or more or having a corresponding volatility under the particular conditions of use” [3]. Additionally, the quite dated—although still highly cited in the literature [4]—1989 World Health Organization’s (WHO) definition classifies as a VOC any organic chemical having a boiling point up to 250 °C measured at a standard atmospheric pressure of 101.3 kPa. Based on this definition, the WHO subdivided VOCs into different classes: very volatile organic compounds, VVOCs, having boiling points ranging from <0 °C to 50–100 °C, such as propane (C_3_H_8_), butane (C_4_H_10_), methyl chloride (CH_3_Cl); and volatile organic compounds, VOCs, with boiling points in the range from 50–100 °C to 240–260 °C, including substances such as formaldehyde (CH_2_O), limonene (C_10_H_16_), and ethanol (C_2_H_5_OH). The WHO also defined an additional category of semi-volatile organic compounds, SVOCs, including substances having boiling points ranging from 240–260 °C to 380–400 °C, such as some pesticides like dichlorodiphenyltrichloroethane (DDT), chlordane or some plasticizers like phthalates [5].

**Figure 1:**
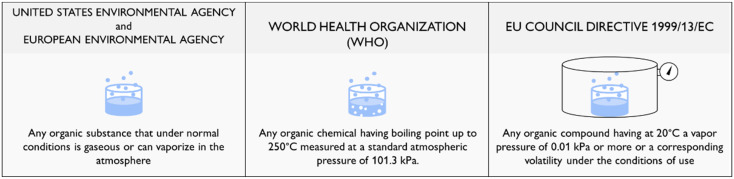
Definition of VOCs according to the United States Environmental Agency (EPA) and the European Environmental Agency (EEA), the World Health Organization (WHO) and the EU Council Directive 1999/13/EC.

Without going deeper into the merits of the diverse definitions of VOCs, schematically summarized into Figure 1, it is quite glaring that all of them align in proving the abundance of organic chemicals identifiable as volatile in many different types of environments.

Benzene (C_6_H_6_), toluene (C_7_H_8_), ethyl benzene (C_8_H_10_), ortho-, meta- and para-xylene, (known as BTEX) (C_8_H_10_), acetone (C_3_H_6_O), styrene (C_8_H_8_), and benzyl alcohol (C_7_H_8_O), are just a few examples of commonly known organic substances having vapor pressure values higher than 0.01 kPa at 20 °C and/or boiling points below 250 °C, that must therefore be considered as VOCs. These substances may be found in ordinary home indoor sites, and in other countless indoor and outdoor environments (and microenvironments) such as those located in industries [6], commercial places [7], hospitals [8], schools [9], etc. For example, among the most diffused VOCs in homes, during the analysis of the inner air of 5000 houses in Japan, acetaldehyde (C_2_H_4_O), toluene, and formaldehyde were found to be the most abundant VOCs [10]. In another study, the analysis of the inner air in art and craft rooms as well as in common class rooms in a primary school showed mainly the presence of benzyl alcohol, styrene, toluene, ethylbenzene (C_8_H_10_), and xylene [11]. 

In general, VOCs may be emitted from countless sources, such as furnishing items, building materials, lavatory and laundry products, and biological matter (such as food), etc. [12,13]. For instance, the presence has been observed of a considerably high amount of toxic formaldehyde in a sealed room containing commonly employed, medium-density fiberboards [14], and a sensibly increased concentration of toluene was proved in kitchens during dishwasher washing cycles [15]. 

Different environments imply the presence of different VOCs, and which varieties and their corresponding concentrations are not only determined and influenced by the materials from which they are emitted, but also from the atmospheric conditions, such as temperature or relative humidity [16], the presence of other materials which may act as adsorbers of VOCs [17,18], the rate of air flux/ventilation [19], and the presence and intensity of visible light/UV irradiation [20], etc. Thus, it is not possible to tabulate general average concentration values of VOCs in the function of similar environments; however, based on numerous studies reported in the literature, it is achievable to draw up lists of VOCs more likely emitted from specific sources and materials in determined situations [21,22]. For example, besides the recognizable emission of VOCs in chemical industries traceable to the mere pure substances [23], it is well known that cellulosic materials such as wood or paper emit acetic and formic acid due to the hydrolysis of acetyl group esters in hemicellulose [24]. Additionally, a large number of polymeric materials used in consumer goods such as furnishings [25], artificial leather or building materials, emit certain VOCs. Bis(2-ethylhexyl)phthalate (DEHP), a plasticizer with significant health concerns, is emitted from poly(vinyl chloride) (PVC) [26], while styrene, recognized as cancerogenic, is emitted from degraded polystyrene (PS) [27].

Cities and high-traffic areas are especially polluted by VOCs emitted from the use of motor vehicle fuels (considering both fuel evaporation and exhaust gas) [28,29], such as toluene, benzene or heptane (C_7_H_16_) [30].

Some specific VOCs are also emitted during food processing as well as during food degradation while 1-butanol (C_4_H_10_O), 1-hexanol (C_6_H_14_O), 2-ethyl-hexanol (C_8_H_18_O) and some volatile fatty acids, such as butyric (C_4_H_8_O_2_), valeric (C_5_H_10_O_2_) or caproic (C_6_H_12_O_2_) acids, are produced during the spoilage of meat, fish, or fruit, or more generally during the decomposition, i.e., anaerobic digestion, of biomass [31]. 

Many plants and flowers also emit specific VOCs. Actually, phytogenic volatile organic compounds (PVOCs) represent the most abundant VOCs present in the atmosphere [32]. What we recognize as natural perfumes and fragrances capable of stimulating our senses causing an upsurge of sensations and feelings, are nothing but VOCs. For example, cinnamyl alcohol (C_9_H_10_O), having an intense smell of sweet hyacinth with balsamic and spicy notes, is a VOC found in cinnamon leaves and flowers [33]. Citronellol (C_10_H_20_O), smelling rosy, sweet and of citrus, is a monoterpenoid VOC principally found in roses and pelargonium flowers [34]. These substances are mainly released by flowers to attract pollinators, while other natural VOCs, such as isoprenoids, are naturally released by plants to improve resistance in response to abiotic stresses [35,36]. 

Many other biological and microbiological processes also imply the release of characteristic VOCs [37,38]. Among them, VOCs emitted by microorganisms (i.e., bacteria, archaea, fungi, and protists) are specifically classified as Microbial Volatile Organic Compounds (MVOCs) and comprise a large variety of chemicals such as fatty acids and their derivatives, nitrogen- and sulfur-containing compounds, aromatics and terpenoids [39,40]. Other VOCs are emitted in the biological processes occurring in human bodies [41,42,43]. For example, it has been observed that breath samples from breast cancer patients contain a unique combination of hydrocarbons, such as alkanes and monomethylated alkanes [44,45].

Hundreds of different VOCs are thus diffused and present in an infinite number of environments whether deriving from degradation processes, biological processes, natural events, or human activities such as industrial productions, transportation, etc. Consequently, the detection and quantification of VOCs are tactical to investigate the interactions of the volatile chemicals with the surrounding environments as well as to determine and study the emission sources. Table 1 reports some of the most common VOCs and their typical emission sources.

Classic methods for the analysis of VOCs are gas and liquid chromatography (GC, LC, HPCL, etc.), whether coupled with other techniques such as mass spectroscopy (MS), time of flight (TOF), thermal desorption (TD), or olfactometric detection (e.g., GC-O), etc. [46,47,48]. Other techniques include, for example, selected-ion flow-tube mass spectrometry (SIFT-MS) or proton-transfer-reaction mass spectrometry (PTR-MS) [49,50]. The analysis of VOCs may be carried out directly injecting the air to be analyzed into the instrument (e.g., headspace analysis) or by firstly adsorbing VOCs on passive or active samplers thus desorbing them in the selected mobile phase for analysis (such as in the case of ion chromatography). These techniques are certainly highly sensitive and efficient but are expensive and energy/time-consuming. In most of the cases they are also not portable, with important drawbacks [51], while the few commercially available portable tools for VOCs analysis are poorly efficient, have high LOD and are not selective to specific VOCs, such as in the case of a photoionization detector (PID) [52].

In the past decades the literature has reported novel VOC sensors and biosensors designed for solving these issues with remarkable results, as reported in different reviews and research papers [53,54,55,56,57]. In general, VOC sensors are devices capable of registering electrical, photophysical, mechanical, or biological changes, after the interaction with specific volatile compounds. These changes are converted into signals, of which the intensity normally depends on analyte concentrations, or analyte chemical and physical characteristics [58]. Among all sensors, the subclass of biosensors indicates sensors containing a biological recognition element, whether that be enzymes, proteins, antibodies, nucleic acids, cells, tissues or receptors, that interact with the VOCs [59,60,61,62].

VOC sensors and biosensors have emerged as alternatives to classic analytical tools mainly due to their faster response, cheaper analysis, and portable characteristics, while other features include enhanced selectivity, lower power consumption, or more rapid recovery times. VOC sensors and biosensors have been successfully employed in a large number of applications in food safety analysis, environmental monitoring, clinical analysis and medical diagnosis [63,64,65,66]; however, it must be highlighted that the majority of sensors and biosensors reported in previous years were developed without, or by poorly considering any green and sustainable characteristics of the final devices or of the production processes.

Recently, and more specifically in the last couple of years, different national and international policies have started firmly pushing for a sustainable development and a green transition [67,68,69,70,71,72,73]. For example, the European Green Deal aims at “making Europe climate neutral by 2050, by boosting the economy through green technology, by creating sustainable industry and transport, and by cutting pollution” [74]. All these policies directly influences any sort of R&D and R&I activity [75,76,77,78], including the design of novel VOC sensors and biosensors [79].

From this perspective, a review on the most innovative VOC sensors and biosensors recently developed with environmentally friendly and sustainable characteristics is herein reported, integrating the current reviews present in the literature in the field of VOC sensors and biosensors [80,81,82,83,84,85,86,87]. The review highlights recent trends in the research of green approaches to substitute and replace classic poorly sustainable sensors, in line and accordance with the most recent environmental policies and researchers’ ethical spirt of sustainable growth. These approaches include manufacturing processes carried out using biomass and waste derived materials, the use of abundant elements in place of rare metals, the design of low energy consuming methods or the exploitation of biological activities, exploiting innovative technologies such as printed electronics, nanotechnology, silicon photonics, or biotechnology [88,89,90].

The sensors and biosensors herein reported include tools for the direct analysis of air, as well as systems for the detection of VOCs adsorbed and redispersed—using the already cited passive or active samplers—in aqueous solutions (such as electrochemical devices).

The article is presented in a logical form to be informative and pedagogic for anyone looking for a deeper understanding of the topic. The review is divided into three different sections presenting VOC sensors and biosensors in the function of highly captivating applications, including environmental analysis, intelligent food packaging design, and medical diagnosis, making the manuscript attractive for both readers having expertise in the field but also for anyone with no specific knowledge who wants to explore the matter.

**Figure 2:**
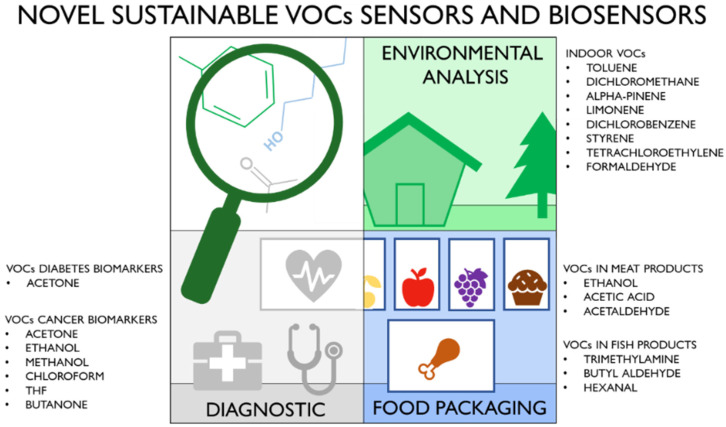
Field of applications of the novel sustainable sensors and biosensors and most relevant analyte VOCs reported in the present review.

In detail, Section 1 includes sensors and biosensors for environmental analysis, especially focusing on VOCs found in common indoor environments. Section 2 describes VOC sensors and biosensors for food packaging applications, where the detection of VOCs is crucial to understanding the freshness of food and the presence of possible active degradation processes. Section 3 is focused on sensors and biosensors for medical uses, of which the applicability can lead to diagnosing diseases easily and quickly. Each Section firstly discusses the most important VOCs found in the specific field and related challenges, thus, the most recent works on the preparation of sensors and biosensors with green characteristics are reported. The conclusion describes perspectives and challenges for future developments. Figure 2 summarizes the sensors and biosensors for specific VOCs’ detection described in each section.

## 2. Section A: Environmental Analysis

The environmental analysis of VOCs aims at the detection and quantification of organic compounds that might involve any biological interaction, including human health issues, plant defense mechanisms, animal toxicity concerns, etc. Without considering particular environments or situations, such as the analysis of gas leaching in pipes or in reactors (which can be undertaken, due to extremely high concentrations of VOCs, using low sensitive sensors and tools), the environmental analysis of VOCs is generally related to the selective detection of common indoor pollutants at low concentrations. Many VOCs are indeed classified as toxic and might cause asthma and other respiratory symptoms/diseases, headaches, nausea, or more severe problems such as convulsions and comas [91]. Some VOCs are also recognized as carcinogenic, especially targeting the liver, kidneys, brain, and nervous system [92]. Therefore, the analysis of VOCs in indoor environments is crucial to determine eventual chronic exposition to toxic chemicals and to avoid severe health issues. In this view, the development of sustainable sensors and biosensors for indoor pollutants has gained much interest especially addressing the current directives of sustainable R&D.

It has been calculated that normally a person spends almost 80% of its life in indoor environments. Thus, a special focus of environmental analysis is the determination and quantification of VOCs in spaces generally occupied during a day such as homes, offices, schools, classrooms, vehicles, and stores [93,94]. VOCs found in these environments are mainly emitted from sources such as construction materials, furnishing, paints, glues, heating appliances, tobacco smoke, cooking, and cleaning products [95,96]. Due to the impossibility of tabulating general concentration values in indoor environments, Table 2 reports the most diffused VOCs in houses and in a primary school and their maximum concentrations.

Among all VOCs present in these types of environments, researchers’ efforts of recent years have specifically focused on the development of greener and more sustainable sensors and biosensors especially aimed at the detection of toluene, dichloromethane, limonene, dichlorobenzene, styrene, tetrachloroethylene, and formaldehyde. 

### 2.1. Detection of Toluene

Toluene (C_7_H_8_) is an aromatic compound used in the manufacturing of many goods such as foams for furniture and insulation materials, coatings, or shoes. It has a time weighted average (TWA) of 20 ppm (8 h) and its vapor might irritate the skin, eyes, and the mucous membranes of the throat, possibly causing headache, vertigo, or fatigue [98]. 

Wang et al. [99] prepared an inexpensive sensor for the detection of toluene based on Fe, one of the most abundant elements in the Earth’s crust, and Ni, a metal having important recyclability properties [75,100]. The sensor, in the form of mesoporous NiFe_2_O_4_, was synthesized through a solvent-free simple method producing limited quantities of waste. The sensor had a framework thickness ranging from 8.5 to 5 nm and a specific surface area ranging from 134 to 216 m^2^ g^−1^. During the testing for gas detection, it was proved that the mesoporous NiFe_2_O_4_ with both an ultrathin framework and large specific surface area could detect toluene in concentrations ranging up to 1000 ppb, showing that the response, selectivity, and stability were remarkably enhanced with respect to commonly employed NiFe-based sensors.

In previous years, different lanthanide complexes have been reported as simple, sensitive, and inexpensive analytical tools for the determination of many organic solvents, metal ions and in general gases due to their structural and unique luminescent properties. Very recently, they have been also proved to be usable as sustainable sensors for the specific detection of toluene [101]. In details a new sequence of lanthanide metal-organic frameworks (LnMOFs) was prepared though a simple and inexpensive solvothermal reaction, using lanthanide (III) nitrates, methylmalonic acid as the ligand and 1,10-phenanthroline as the capping agent. The luminescence analysis of LnMOFs in the presence of different organic solvents, showed an evident and marked response though the detection of toluene, proving the possible use of LnMOFs as a highly selective luminous sensor for this type of VOC.

Some environmentally friendly carbon dots have been also proposed as possible sensors for organic compounds’ detection. For example, Dong et al. recently reported the preparation of nitrogen and sulfur doped carbon dots as sensors for toluene [102]. Importantly, the materials were prepared using citric acid as the carbon source, sensibly improving the sustainability of the synthetic process, considering that citric acid might be produced by yeasts via biomass valorization [103].

A few years ago, the possibility was proved of preparing a fiber optic enzymatic biosensor featuring cost-effective, real time, continuous, and in situ measurements of toluene. A sensor was prepared using toluene ortho-monooxygenase (TOM) as the biological recognition element, and an optical fiber coated with an oxygen-sensitive ruthenium phosphorescent dye as the transducer [104]. The detection of toluene was carried out based on the enzymatic reaction catalyzed by TOM, which resulted in the consumption of oxygen and, consequently, changes in the phosphorescence intensity.

### 2.2. Detection of Dichloromethane

Dichloromethane (DCM) (CH_2_Cl_2_) is largely used in industry due to its high volatility and ability to dissolve many chemicals and it is used to produce paint removers or adhesives, among others. DCM has a TWA (8 h) of 50 ppm, and its hazardous properties include the irritation of skin and mucous membranes and the cause of headache, vertigo, nausea, vomiting and anemia. It has been classified as likely to be carcinogenic [98].

**Figure 3:**
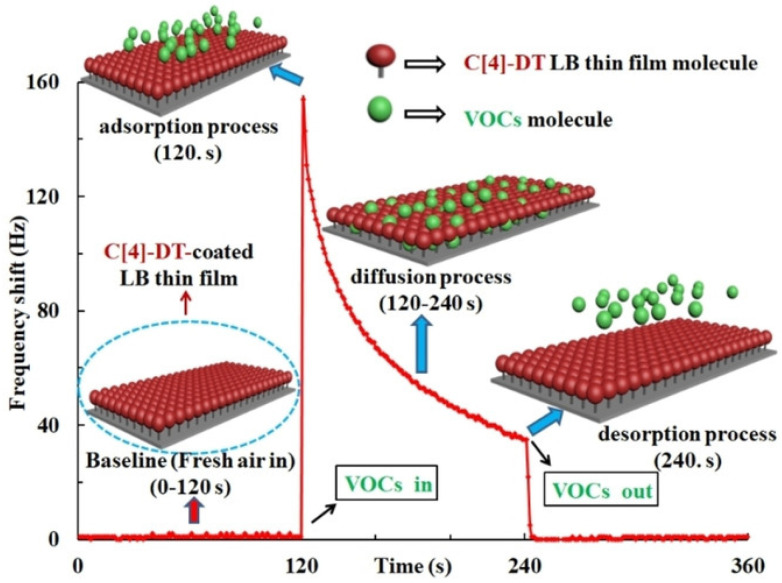
Schematic representation of the interaction of VOCs with the calix[4]arene-dithiourea receptor. Reprinted with permission from ref. [105]. Copyright 2021 Wiley.

In the last decade, the quartz crystal microbalance (QCM) technique combined with a surface plasmon resonance (SPR) system using Langmuir–Blodgett (LB) thin films have emerged for the detection of VOCs due to the high sensitivity and reliability of the methodology combined with low experimental costs and limited environmental impact. Durmaz et al. exploited these features to prepare a sensitive LB film coated QCM sensor for the detection of DCM [105]. In detail, a calix[4]arene-dithiourea receptor, denoted “C[4]-DT”, was used to form a thin film over quartz crystals for QCM measurements. As shown in Figure 3, the so-prepared C[4]-DT LB film-coated QCM sensor was used for the detection of several VOCs.

The system showed a specifically selective response to the DCM rather than other vapors with a limit of quantification of 0.5 ppm. Additionally, the sensor was proved to have a good reproducibility, rapid response time, and excellent full recovery. 

**Figure 4:**
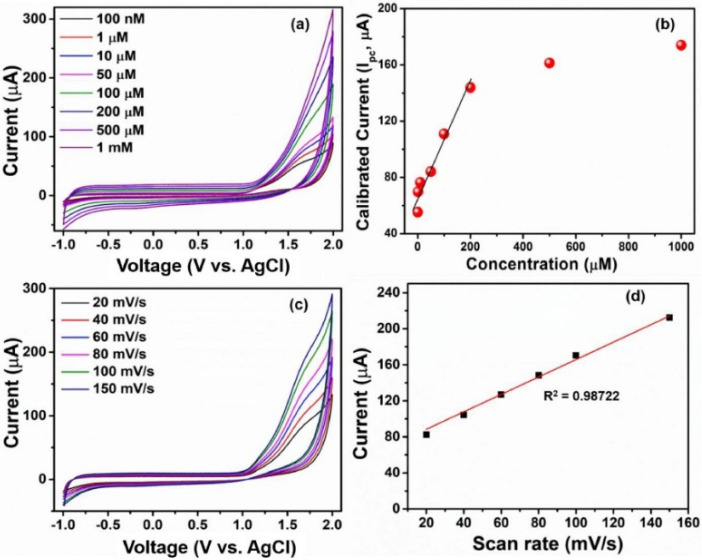
(**a**) CV curves obtained varying the DCM concentrations from 100 nM to 1 mM, (**b**) calibrated current of cathodic peak versus concentration of DCM chemical, (**c**) CV measurements at various scan rates and (**d**) calibrated current at cathodic peak versus scan rate of the hexagonal ZnO NPys modified electrode. Reprinted with permission from ref. [106]. Copyright 2019 Elsevier.

Based on the fact that electrochemical methods for the detection of toxic chemicals are particularly highly sensitive, economic, and portable, Shink et al. proposed an environmentally friendly electrode for the detection of DCM based on a zinc oxide modified disposable screen printed electrode (SPE) [106]. In detail, the authors developed a synthetic methodology to produce hexagonal zinc oxide (ZnO) nanopyramids (NPys), of which the morphology could remarkably improve the performance of the sensor. ZnO NPys were synthesized by a simple and fast hydrothermal procedure using zinc acetate as the precursor and oleylamine as the surfactant. As illustrated in Figure 4, the sensor showed good behavior in the detection of DMC through a series of cyclovoltammetric (CV) analysis.

The modified disposable SPE chemical sensor showed a good sensing behavior for the detection of DCM with high sensitivity, a limit of detection of 17.3 µM and an excellent linearity in the range of ~100 nM to 200 µM.

More recently, another study reported the preparation of a highly sensitive sensor for the detection of DCM based on upconverting nanoparticles (UCNPs) [107]. UCNPs are nanoparticles capable of converting low energy incident photons into emitted photons with higher energy, and have particularly emerged for background-free imaging, biological detection, temperature sensing, and many other applications. The key feature of UCNPs is the possibility of preparing sensors with a high sensitivity and a low detection limit along with the important advantage of low energy consumption. The sensor for the detection of DCM was specifically prepared in the form of NaGdF_4_:Yb,Er@NaYF_4_:Yb active core@shell upconverting nanoparticles (UCNPs) by depositing UCNPs on porous anodic alumina oxide templates supported by glass slides, forming a thin film-like gas sensor. The nanoporous fluorescent sensor was capable of detecting dichloromethane with a detection limit of 2.9 ppm at room temperature.

Different DCM bacteria destructors have also been proved to be suitable for the preparation of sustainable sensors for DCM detection. In detail, *ethylobacteria-Methylobacterium dichloromethanicum* *DM4*, *Methylobacterium extorquens* *DM17*, *Methylopila helvetica DM6*, and *Ancylobacter dichloromethanicus DM16* immobilized on membranes fixed on a pH-sensitive transistor, could interact with DCM leading to a change in the output signal of the transistor [108].

### 2.3. Detection of Limonene and α-Pinene

α-pinene (C_10_H_16_) and limonene (C_10_H_16_) are natural substances mainly found in the oils of coniferous trees (α-pinene) and citrus fruit peels (limonene). α-pinene is principally used to produce perfumes and fragrances and has a TWA (8 h) of 20 ppm. At low concentrations it has therapeutics properties [109], while at high concentration it may cause allergic reactions, and could be highly toxic.

Limonene has a TWA of 30 ppm and its quite safe for human uses although it may cause allergic reactions and toxicity issues by inhalation at high concentrations. Limonene is used as solvent, fragrance, and insecticide [98].

In a similar manner to the detection of DCM, quartz crystal microbalance (QCM) techniques were also exploited for the detection of limonene and α-pinene. In detail, a sensor for the detection of limonene was prepared using a QCM chip as the sensor transducer and ethyl cellulose as the sensing material [110]. The use of ethyl cellulose (EC) is of particular interest since EC is derived from cellulose, i.e., the most renewable natural polymer on Earth [111]. The sensor was specifically proved to detect limonene up to 6000 mg m^−3^, with a limit of detection (LOD) of 300 mg m^−3^. The sensor was also demonstrated to be stable and efficient since it could be used for up to five cycles and for a month before observing significant losses of activity.

**Figure 5:**
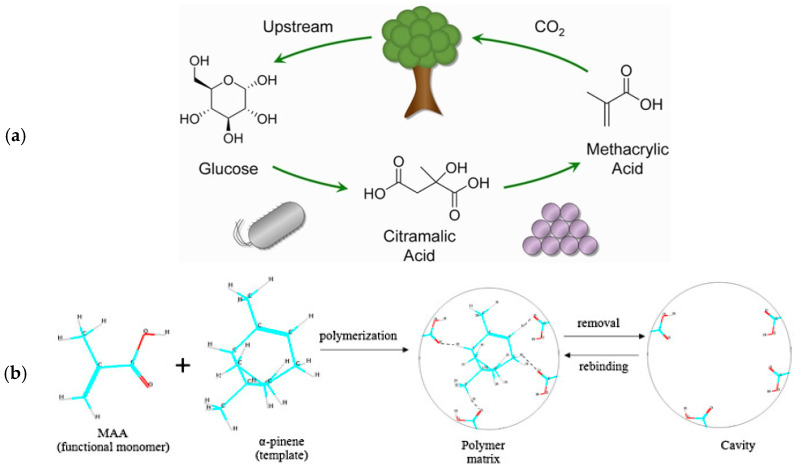
(**a**) Scheme of the hybrid fermentation and thermocatalysis to produce methacrylic acid (MAA) from glucose. Reprinted with permission from ref. [113]. Copyright 2021 American Chemical Society. (**b**) Illustration of molecular imprint polymer (MIP) concept made of MAA. Reprinted with permission from ref. [112]. Copyright 2013 Elsevier.

On the other hand, the detection of α-pinene is quite complicated and few works have reported the successful design of novel sustainable sensors, making the research highly challenging. Among the few outstanding examples, a sensor for the detection of α-pinene was prepared by manufacturing a highly selective molecularly imprinted polymer (MIP) layer combined with an interdigitated electrode (IDE) as a sensor. Importantly, the IED was prepared using methacrylic acid (MAA) as the sensing material [112]. The sensor was proved to be remarkably selective and efficient. Significantly, considering that it has been recently demonstrated that it is possible to produce MAA from biomass-derived glucose, the manufacturing of this sensor can be considered potentially sustainable, as summarized in Figure 5 [113].

### 2.4. Detection of Dichlorobenzene

Dichlorobenzene (DCB) (C_6_H_4_Cl_2_) in its three different isomeric forms (1,2; 1,4 and 1,3) is used in space deodorants, fumigants, insecticides, and herbicides as well as in the synthesis of dyes and resins. The lower value of TWA (8 h) of DCB (corresponding to 1,4-dichlorobenzene) is 25 ppm. Inhalation of the vapor of DCB results in irritation to the eyes, skin, and throat. DCB has also the potential to cause cancer [98].

A few years ago, Chao et al. demonstrated the possibility of producing mesoporous molecular sieves MCM-41 from coal fly ash at room temperature via a green and efficient reaction [114]. MCM-41 is a widely used material with applications in catalysis, separation processes, and adsorption of gases and liquid. This last feature was specifically exploited by Rahman et al. to design a simple, inexpensive, potentially sustainable, consistent, portable, and reliable chemical sensor for 1,2-dichlorobenzene detection [115]. The sensor was fabricated by depositing a thin layer of MCM-41 on a glassy carbon electrode (GCE). The sensor, used through an electrochemical approach, showed good sensitivity and a short response time of 14.0 s, while the linear dynamic range and the detection limit were reported as 0.089 nM to 8.9 mM and 13.0 pM, respectively. 

### 2.5. Detection of Styrene

Styrene (C_8_H_8_) is extensively used in the manufacturing of numerous polymers and copolymers such as polystyrene, acrylonitrile-butadiene-styrene (ABS), styrene-butadiene latex, for the fabrication of different goods including foam packaging, toys, shoes, and furnishings. Styrene has a TWA (8 h) of 20 ppm, and its vapor irritates the eyes and mucous membranes. The inhalation of high concentrations of styrene can cause polyneuritis. It is also reasonably anticipated to be a human carcinogen [98].

**Figure 6:**
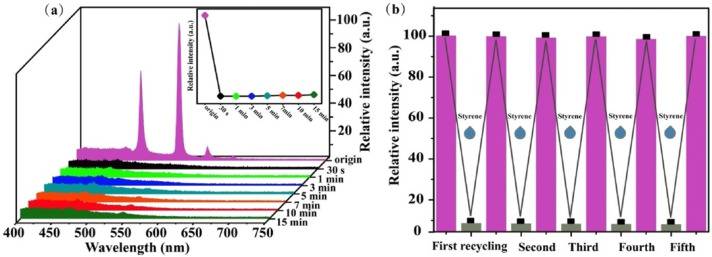
(**a**) Time-dependent emission spectra of the Tb-MOFs film responded to 20 μL styrene vapor; (**b**) Emission intensity of five recyclable experiments of sensing styrene, Reprinted with permission from ref. [116]. Copyright 2020 Elsevier.

Recently, Bi et al. developed a Terbium-based metal-organic frameworks (MOF) for the efficient detection of styrene. Td-MOF (Tb^3+^) was prepared based on an innovative, facile, and low-energy consuming (at room temperature) method [116]. Td-MOF was thus homogeneously embedded into a PVA film and deposited on silica gel sheets, forming a luminescent vapor sensor film for styrene detection. A sequence of photoluminescence (PL) tests demonstrated that Tb-MOFs showed a significant response rate and high sensitivity to styrene vapor. In addition, as shown in Figure 6a, time-dependent fluorescence quenching indicated that the emission of the film was immediately quenched by exposure to styrene vapor (in only 30 s), and the intensity remained unchanged over time, proving an excellent sensitivity performance. Recyclable tests, i.e., by carrying out experiments followed by a drying procedure in an oven, also proved the good reversibility and reusability of the Td-MOF, as illustrated in Figure 6b.

A few years ago the possible utilization of bacteria for the preparation of biosensors for styrene detection was also demonstrated, such as in the case of a biosensor based on the regulation system of the styrene catabolic pathway present in the *Pseudomonas* sp. strain Y2 [117]; however, this type of approach has not been followed up in recent years, although it has tremendous potentialities.

### 2.6. Detection of Tetrachloroethylene

Tetrachloroethylene (C_2_Cl_4_) is principally used as a chemical intermediate and as a solvent in the textile and metal industries. Tetrachloroethylene has a TWA (8 h) of 25 ppm and the exposure to its vapors can cause eye irritation, narcotic action, vertigo, nausea, and headache. Tetrachloroethylene is also suspected to cause cancer [98].

A ZnO-based sensor capable of detecting tetrachloroethylene was recently proposed by Zhao et al. [118]. In detail, the researchers developed a new method for the chip-level pyrolysis of as-grown zeolitic imidazolate framework films to hierarchical and structured ZnO sheets composed of interpenetrated nanometer particles. The tunable introduction of interpenetrated particles generated adjustable oxygen vacancies, modifying the electronic structure of the sensing materials. As a result, the sensors showed improved diffusion, penetration, and adsorption of the relevant gases, resulting in enhanced sensitivity and a shortened response time toward the detection of different VOCs at the ppb-level, including tetrachloroethylene. The facile synthetic approach using a largely available material, i.e., ZnO, made the novel sensor a good candidate for sustainable scaled-up productions and commercialization.

### 2.7. Detection of Formaldehyde

Formaldehyde (CH_2_O) is used in the manufacturing of many different products including adhesives, abrasive materials, insulating materials, coatings, and polyacetal plastics-based materials. In indoor environments it is mostly emitted from building materials. Formaldehyde is a highly toxic chemical with a TWA (8 h) of 0.1 ppm. The inhalation of formaldehyde irritates the mucous membranes, while chronic symptoms include renal and hepatic damage. It is considered cancerogenic [98]. 

**Figure 7:**
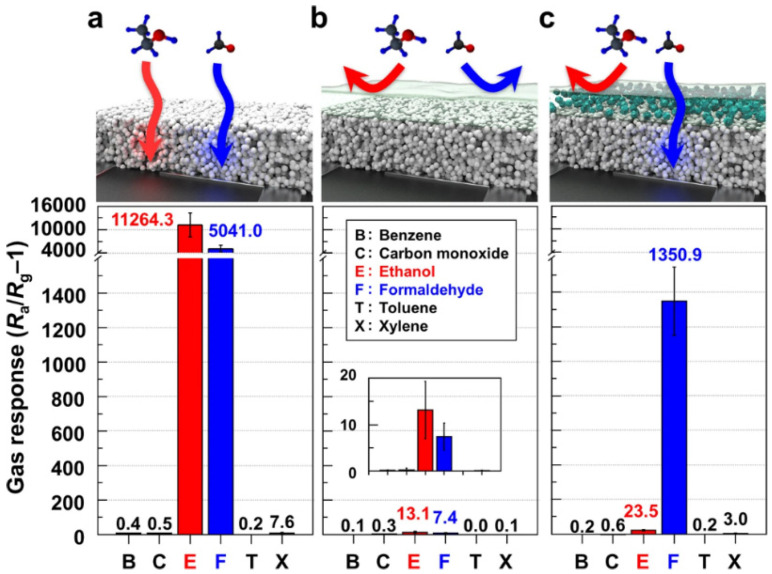
(**a**) Gas responses of a bare TiO_2_, (**b**) Pure PEBA/TiO_2_, and (**c**) 5MMM/TiO_2_ sensors exposed to 5 ppm benzene, carbon dioxide, ethanol, formaldehyde, toluene, and p-xylene at 23 °C under UV illumination (λ: 365 nm). Error bars represent SD of the mean. Reprinted with permission from ref. [119]. Copyright 2021 Springer Nature.

Recently, Lee et al. reported the manufacturing of a monolithic flexible sensor for the detection of formaldehyde at the ppb-level [119]. The sensor was produced by depositing a TiO_2_ sensing film on a polyethylene terephthalate substrate and by covering the film with an overlayer of molecular sieving a ZIF-7/polyether block amide (mixed matrix membrane, MMM). The sensor was designed to selectively detect formaldehyde by a sensing photoactivation at room temperature. The sensor showed ultrahigh selectivity (response ratio > 50) and response (resistance ratio > 1100) to the exposure at only 5 ppm of formaldehyde. Figure 7 illustrates the selectivity toward the detection of formaldehyde of the novel MMM/TiO_2_ sensor also in the presence of ethanol (normally sensibly affecting the detection of formaldehyde). 

A high-performance formaldehyde sensor was prepared by a surface micro-fabrication technique depositing a LaFeO_3_ (LFO) thin film on a silica substrate [120]. The sensing performances demonstrated that the novel formaldehyde sensors had a remarkable sensitive response and low detection limit toward the ppb-level. In detail, the sensor exhibited a detection limit of 50 ppb and outstanding replicability with a maximum drift of the baseline resistance from different batches of the sensor gas sensors of only 5.4%, and the maximum drift of the response value of 6.5%. In addition, the response values of the sensors remained stable for up to 18 days, with an absolute deviation of response value of approximatively 0.04.

Other recent sustainable approaches for the preparation of sensors for formaldehyde detection include the use of largely available and inexpensive materials such as tin and zinc [121,122,123,124,125,126,127,128], the second most abundant element in the Earth’s crusts, i.e., silicon [129,130], the use of biomass-derived materials, such as bacterial cellulose [131] or egg-white [132]. 

A biosensor based on formaldehyde dehydrogenase and chitosan has also been recently reported [133]. The sensor was prepared through a low-cost inkjet printing technology by depositing a polyion-complex of FDH and chitosan on an electrode connected with an organic field-effect transistor. The biosensor could detect formaldehyde with an LOD of 3.1 μM in aqueous solution.

## 3. Section B: Food Packaging

The demands of the users (food producers, food processors, logistic operators, distributors, and consumers) in the food industry sector are increasing in terms of food safety, quality, and traceability [134]. Throughout the food chain (production, storage, transport, and sale) there are a wide variety of factors (microorganisms, enzymes, temperature, etc.), that can corrupt food products and reduce their shelf life. This is the reason why, in particular, food packaging plays a key role in maintaining the quality of food as well as preserving it from contamination [135]. Traditional packaging systems merely isolate food from the external environment without providing information on the freshness or condition of the food beyond the expiration date. Thus, it is constantly necessary to innovate in the field of food packaging, not only to reduce its environmental footprint, but also to increase its functions. In this scenario arises intelligent packaging, a new packaging technology that integrates traditional packaging systems with intelligent functionalities, including the monitoring of changes in the food product, as well as quality and safety information [136,137], by temperature, humidity, pH, and light exposure measurements [138,139,140,141], or through the detection of specific VOCs [134,142,143,144,145]. For example, 1-butanol (C_4_H_10_O), 1-hexanol (C_6_H_14_O), 2-ethyl-hexanol (C_8_H_18_O), 1-octen-3-ol (C_8_H_16_O), butanal (C_4_H_8_O), hexanal (C_6_H_12_O) and nonanal (C_9_H_18_O), which are indicators of freshness in food products, while other VOCs, such as fatty volatile acids, are produced during the spoilage of foods [146]. 

When it comes to incorporating sensing technologies into food packaging materials, the industry trend is to do so for meat or fish products [147].

### 3.1. VOCs Detection in Meat Products

Microbial growth, oxidation and enzymatic autolysis are the three main mechanisms of meat deterioration. During meat spoilage, proteins and lipids decompose to form new compounds that negatively affect product quality. The intrinsic factors related to meat spoilage include pH, water activity and nutrient content of the meat, while extrinsic factors include temperature and atmospheric conditions surrounding the product [148]. For example, when microbial spoilage occurs, there is a decrease in pH due to the release of lactic acid. The microbes commonly associated with this phenomenon are of the genus *Pseudomonas* and a traditional sensor/biosensor should detect specific *Pseudomonas* presence by antigen/antibody reactions or similar [149]. Since microbial spoilage may not occur homogeneously throughout the meat product and the detection of these bacteria would require the sensor to be in direct contact with the entire product, it is most desirable that the target product detected by the sensor be a gaseous by-product released into the packaging space. Under normal packaging conditions, several metabolites are formed in the packaging space including CO_2_, O_2_, volatile nitrogen compounds and biogenic amines. As far as this review is concerned, it should be mentioned that the most common VOCs released during meat spoilage are alcohols, phenols, ketones, acids and sulfur-containing compounds [150]. 

Regarding the detection of VOCs in the meat industry, the most common trends have been towards the detection of alcohols or acetic acid. This is because alcohols such as 3-methyl-1-butanol (C_5_H_12_O) or 1-hexanol (C_6_H_14_O) are indicative of *Salmonella* contamination in packaged beef, while acetic acid is an indicator of microbial population growth. Hence, Sankaran et al., elaborated olfactory bio-derived sensors mimicking insect odorant binding protein to detect them in low concentrations at room temperature. These were biosensors based on quartz crystal microbalance (QMC) with synthetic peptides. This peptide sequence acting as the sensing material was derived from the amino acid sequence of the LUSH protein from *Drosophila* odorant binding protein and can detect alcohols with estimated lower detection limits of <5 ppm [151,152]. On the other hand, in order to be able to detect acetic acid even at low concentrations (1–3 ppm), Panigrahi et al., prepared quartz crystal microbalance (QMC) sensors deposited over synthetic polypeptide [153]. Recently, Han developed a new gas sensor employing ZnO foam as the sensing material aimed at acetic acid with superior sensing performances [154]. 

**Figure 8:**
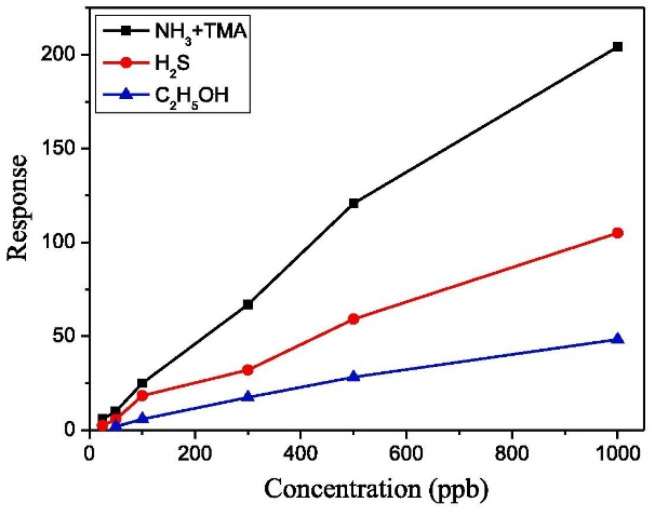
Response curve of Au patch electrode Ag-SnO_2_/SiO_2_/Si metal-insulator-semiconductor capacitive gas sensor for increasing concentrations of ammonia and trimethylamine (NH_3_ + TMA), hydrogen sulfide (H_2_S) and ethanol, Reprinted with permission from ref. [155]. Copyright 2020 Elsevier.

The latest advances in the development of sensors for the detection of alcohols in packaged meat concerned the detection of ethanol (C_2_H_5_OH). Senapati and Sahu prepared an Au patch electrode Ag-SnO_2_/SiO_2_/Si metal-insulator-semiconductor capacitive gas sensor with a high sensitivity (10 ppm) for chicken meat samples [155]. The sensor was prepared using a considerably high amount of inexpensive and largely available Sn and Si, although, it is worth mentioning that the response of these sensors to ethanol is lower than to other gases such as ammonia and trimethylamine or hydrogen sulfide, as shown in Figure 8. 

**Figure 9:**
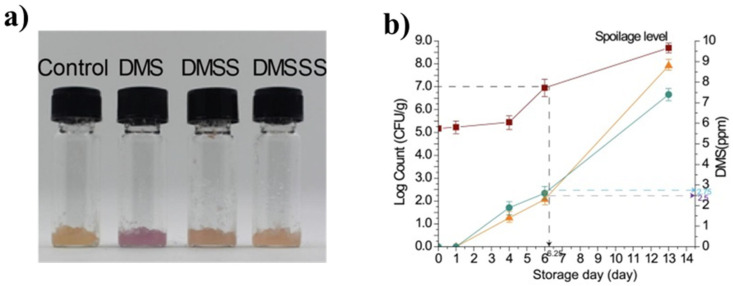
(**a**) Naked-eye sensing response of solid-supported chemosensor toward DMS; (**b**) changes in microbial counts (brown line) and DMS concentration measured by UV-Vis (green line) and GC-MS (orange line) for beef samples stored at 4 °C. Reprinted with permission from ref. [158]. Copyright 2019 Elsevier.

In recent years, the detection of other VOCs related to meat spoilage has also been studied. Acetaldehyde (C_2_H_4_O), resulting from ethanol metabolism, is one of the most important compounds to consider in sophisticated packaging systems. This compound is classified as carcinogenic, and its TWA (8 h) is 25 ppm [156]. It is therefore important to be able to detect this compound quickly and efficiently. Kim et al. fabricated a surface acoustic wave (SAW) sensor that evaluated the storage time of chicken meat (up to 15 days) as a function of increasing acetaldehyde concentration. These authors verified the feasibility of PDMS polymer composite sensors coated with a layer of the SAW device for the detection of aldehyde gas with a 0.989 coefficient of determination between the gas and storage time of chicken meat [157]. Lastly, another VOC released during the spoilage of meat products, and thus acting as a marker, is dimethyl sulfide (DMS, C_2_H_6_S). For its detection, Chow developed environmentally friendly chemosensors based on bimetallic donor–acceptor ensembles (BmDAE) with a selectivity toward DMS 1.0 ppm in real beef samples. This selectivity was clearly observable to the naked eye, since the chemosensor only turned pink in the presence of DMS (Figure 9a). Moreover, the chemosensor response was correlated with the microbial growth level and the storage time, as shown in Figure 9b [158].

### 3.2. VOCs Detection in Fish Products

The consumption of fish or fish-based products is booming due to their health benefits; however, these products are extremely perishable, so it is necessary to develop non-invasive techniques that allow the freshness of the food to be known in more detail rather than just the packaging date. As with meat products, certain VOCs produced by microbial, enzymatic, or autolytic activities during fish spoilage have been identified [159]. Therefore, developing sensors for detecting these compounds is a promising approach.

**Figure 10:**
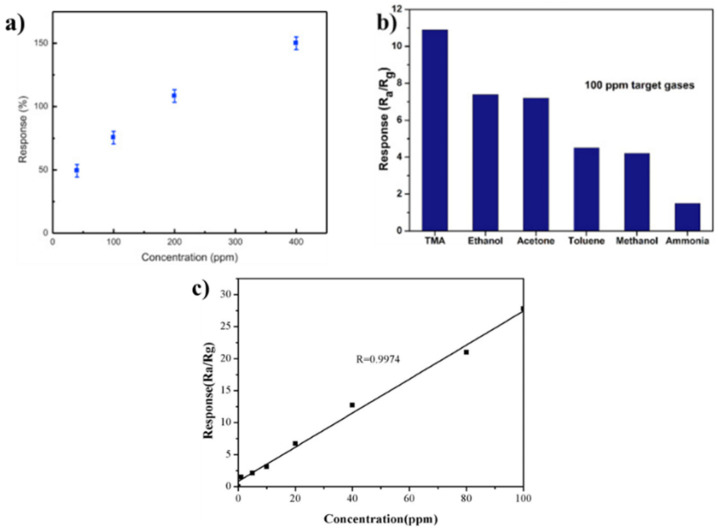
(**a**) TiO_2_ nanotubes sensor response to increasing TMA concentrations (40–400 ppm). Reprinted with permission from ref. [160]. Copyright 2016 Elsevier. (**b**) Response of snowflake-like α-Fe_2_O_3_ hierarchical architectures toward 100 ppm of various testing gases Reprinted with permission from ref. [161]. Copyright 2017 Elsevier. (**c**) Response of α-Fe_2_O_3_ sensor to increasing concentrations of TMA gas (1–100 ppm) Reprinted with permission from ref. [162]. Copyright 2020 Frontiers Media SA.

One of the most characteristic VOCs released during fish spoilage is trimethylamine (TMA, C_3_H_9_N), a chemical produced through the decomposition of proteins, carbohydrates, and fats. Recently, Perillo and Rodríguez employed TiO_2_ membrane nanotubes supported on a flexible substrate as a sensor for TMA detection. This sensor was developed using a simple electrochemical anodization and was able to detect TMA at low temperatures in a very wide detection range (40–400 ppm, Figure 10a) [160]. Importantly, TiO_2_ is a largely available oxide with a very low impact on human health. Other types of sensors that can be used in the detection of TMA in canned fish are those reported by Yang et al. In this case, the authors employed α-Fe_2_O_3_ snowflake-like hierarchical architectures as a TMA gas sensor. The sensors showed an ultra-fast response of 0.9 and 1.5 s for response time and recovery time, respectively, for TMA and other testing gases such as ethanol, acetone, toluene, methanol and ammonia with a sensitivity of 100 ppm, as illustrated in Figure 10b [161]. Along the same lines, Liu et al., (2020) incorporated α-Fe_2_O_3_ nanoparticles in thick films for the detection of TMA in fish. These sensors showed very good selectivity and high sensitivity for TMA with a minimum detection of 1 ppm, as illustrated in Figure 10c [162]. This same metal oxide has been employed by Shen et al. for the development of α- Fe_2_O_3_ modified Au@Pt bimetallic hollow nanocube sensors. These sensors showed a very fast response time (5 s) towards 100 ppm TMA in *Larimichthys crocea* [163]. All these approaches followed the idea of exploiting an abundant element, i.e., Fe, of which its sustainable use has been already discussed.

**Figure 11:**
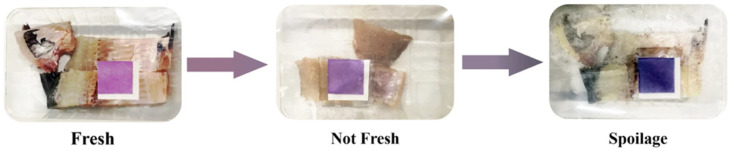
Color change of printable colorimetric paper sensor during monitoring of the freshness of the grass carp within 24 h at 25 °C by Sun et al., Reprinted with permission from ref. [165]. Copyright 2021 Springer Nature.

TMA detection can be also carried out by colorimetric changes. Lv et al. laid the groundwork for the reaction mechanism of a set of colorimetric sensors that included chromogenic materials sensitive to TMA during the deterioration of packaged fresh mackerel. The authors selected six types of metalloporphyrins and tetraphenyl porphyrins (TPP) and showed that MnTPP, NiTPP and FeTPP had the best binding capacity to TMA. Thus, metal porphyrins can be employed for the construction of colorimetric sensors for TMA [164]. Meanwhile, Sun et al. developed a colorimetric printed freshness indicator for fish in modified atmosphere packaging (MAP) [165]. These authors prepared a printable ink based on a natural purple cabbage pigment—which can be potentially also extracted form waste cabbage [166]—carboxymethyl cellulose and glycerin, screen printed it on paper and applied it to grass carp MAP. This label darkens as the TMA content in the fish sample increases as an indicator of spoilage, as shown in Figure 11. The freshness of fish can also be measured non-destructively using fluorescent films. Lai et al. developed highly emissive amorphous tetraphenylethylene (TPEBA) nanoparticles capable of detecting TMA with a detection limit of 0.89 ppm in butterfish [167]. Finally, the most recent advance in the detection of TMA in fish has been the one proposed by Praoboon et al. [168]. The authors developed a paper-based electrochemiluminescence device for the estimation of TMA concentration in freshwater and marine fish samples (red tilapia, yellow tail, salmon, tuna, and catfish). The key to these sensors lay in the fast response they provided (2 min) for a TMA concentration range from 1 × 10^−12^ to 1 × 10^−6^ M.

Although to a lesser extent than the TMA, aldehydes such as hexanal (C_6_H_12_O), octanal (C_8_H_16_O) and nonanal (C_9_H_18_O) are also released from fish products such as grass carp or hairtail fish. In this sense, Jia et al. developed a predictive model to determine the freshness of salmon during cold storage. The authors employed electronic nose with principal component analysis (PCA) and radial basis function neural networks (RFBNN). This system allowed the detection of VOCs such as butyl aldehyde (C_4_H_8_O), amyl aldehyde, hexanal, heptanal (C_7_H_14_O), 1-propanol (C_3_H_8_O), and 1,2-butanone amyl alcohol, which increased proportionally with the level of salmon spoilage [169]. Lastly, Chen et al. prepared a quartz crystal microbalance (QMC) gas sensor modified with the hydrophobic amino-functionalized graphene oxide (AGO) nanocomposite for aldehydes detection in grass carp fish fillets and hairtail fillets. These sensors responded towards aldehydes within 45 ppm under 80% relative humidity during refrigerated storage at 4 °C [170].

## 4. Section C: Diagnostic

As estimated by the World Health Organization [171], every year 12 million global deaths (nearly 25% of total deaths) are attributable to unhealthy environments. Environmental hazards, in particular water, air, and soil pollution, causes hundreds of diseases and health problems. In addition, the WHO has pointed out that two-thirds of the total deaths related to unhealthy environments come from noncommunicable diseases (NCD) such as heart diseases, autoimmune diseases, diabetes, strokes, cancers, and others. The same institution reported that yearly about eight million people die due to the delayed diagnosis of NCD.

An effective strategy to prevent these deaths is the development of devices allowing an early diagnosis of the diseases. The accurate identification and quantification of VOCs emitted from the body can indeed provide information on health and metabolic pathological conditions. In particular, VOC sensors have gained considerable interest for the selective and continuous diagnosis of various physiological and pathological states acting as biomarkers for the identification of numerous diseases in a non-invasive way [172,173,174,175]. Indeed, the key factor of this type of analysis is the detection of VOCs in the exhaled breath of patients through simple, efficient, and inexpensive tools [176,177,178]. For example, some VOCs such as acetone, benzene, ethanol, and isoprene are related to specific diseases and could be used as biomarkers of diabetes, genetic disorders, infectious, cancerous, or renal diseases [75,179,180]. 

In recent years, scientific efforts have especially focused on the design of environmentally friendly sensors and biosensors for the sustainable diagnosis of cancer and diabetes. Moreover, some remarkable results have been also obtained in the diagnosis of asthma, chronic obstructive pulmonary disease, cystic fibrosis, liver cirrhosis and tuberculosis [181,182,183,184,185].

### 4.1. Diabetes Diagnosis

The traditional method for checking diabetes involves collecting blood samples. This type of analysis is precise and accurate but painful, expensive, and invasive. Alternatively, it has been demonstrated that diabetes can be diagnosticated in a non-invasive way by detecting different gaseous VOCs in breath samples. Indeed, the concentrations of olfactory markers of the breath in diabetic patients show significant differences compared to those of healthy patients. For example, acetone (CH_3_COCH_3_) is one the most studied and recognizable VOCs for diabetes diagnosis [186], considering that acetone concentration in diabetic patients is higher than 1.8 ppm [187,188]. 

Ma et al. [189] developed a sensor for acetone detection based on Ni, a metal having important recyclability properties, and Fe, one of the most abundant chemical elements in the Earth’s crust. Porous NiFe_2_O_4_ microspheres were synthetized using an easy procedure, combining a solvothermal step with a heating annealing methodology. As proved by experimental tests, the gas sensors showed a high response to 100 ppm acetone, a low detection limit (200 ppb) and excellent reusability.

A high-performant NiO/SnO_2_ acetone sensor was also prepared via a facile hydrothermal protocol [190]. The gas sensor exhibited improved performances compared to pure tin oxide and showed a fast response, low detection limit (10 ppb) and good selectivity. Similarly, a SnO_2_/ZnO-based sensor able to detect acetone was recently proposed by Dong et al. [191]. In detail, an electrospinning step and a low temperature water bath method was developed for designing SnO_2_/ZnO hetero nanofibers. The sensor was tested with an acetone concentration range of 1 to 100 ppm. The results demonstrated that SnO_2_/ZnO materials exhibited fast response values, and a remarkable, high selectivity to acetone.

A few years ago, Zhang et al. reported a one-step route to prepare C_3_N_4_-SnO_2_ nanocomposites with an outstanding acetone sensing performance [192]. C_3_N_4_ and SnO_2_ are eco-friendly, economic, and easy-to-prepare materials, and the synthetic procedure reported by the researchers was simple, repeatable, and operable. The sensors exhibited about a 20 times improvement of the response sensitivity as well as remarkable selectivity, fast response and repeatability compared with pure tin oxide. The detection limit of 67 ppb was remarkably below the acetone content of diabetes patients’ exhaled breath.

Recently, ZnFe_2_O_4_ has also attracted considerable interest due to its environmentally friendly characteristics, low cost, and excellent stability. Huang et al. designed ZnFe_2_O_4_ nanorods through an easy hydrothermal route [193] with a high gas response of acetone. 

Another study reported the microwave-assisted synthesis of a sensor for the detection of an acetone based on a Co_3_O_4_/rGO nanocomposite [194]. Microwave (MW) irradiation is recognized as a time-saving heating method with remarkable environmentally friendly characteristics such as minimized heating loss and improved energy efficiency [75,195,196]. The tests showed that the materials achieved remarkable response to acetone (0.5~200 ppm) and good selectivity against the gases of hydrogen, methane, hydrogen sulphide, formaldehyde, methanol, methoxyethane and ethanol.

### 4.2. Cancer Diagnosis

Commonly used methodologies for cancer diagnosis implies bronchoscopy and diagnostic imaging (CT scan). These analyses entail some drawbacks such as weak sensitivity or the use of expensive tools. Moreover, bronchoscopy involves anesthesia, which is sometimes correlated with trauma and complications. In the past decade, the detection of specific VOC biomarkers has been identified as a new frontier for non-invasive cancer diagnosis [197]. In detail, VOCs such as toluene, benzene, styrene, ethanol, methanol, acetaldehyde, formaldehyde, and octanal are present in the breath of people suffering cancer [198] in concentrations higher with respect to the health subject [199]. 

Recently, Feller et al. presented the design of a biobased carbon nanorods VOC sensor for the effective detection of acetone, ethanol, and methanol for the early diagnosis of cancer [200]. Importantly, the device was prepared via an easy, fast and green approach through the pyrolysis of a renewable carbon source, i.e., castor oil. 

**Figure 12:**
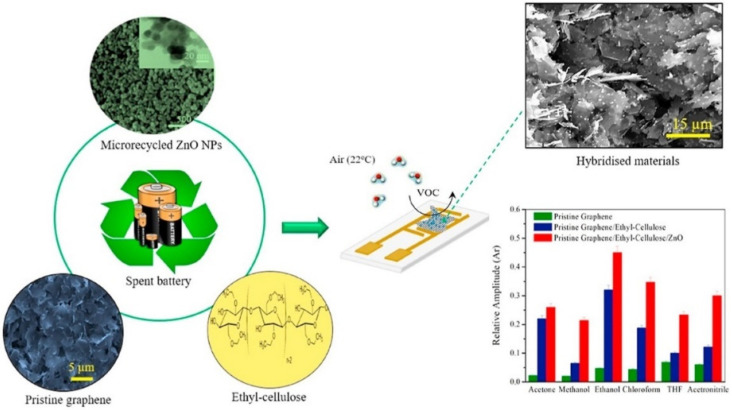
Schematic representation of the preparation of ZnO-based sensors for VOCs detection and cancer diagnosis using spent batteries, Reprinted with permission from ref. [201]. Copyright 2021 Elsevier.

Also Sahajwalla et al. have developed a new sensor with sensing performances tailored for VOC biomarker cancer detection [201]. As illustrated in Figure 12, the tool was synthetized using pristine graphene and zinc oxide nanoparticles recovered from spent Zn–C batteries. 

Preliminary tests showed that the recycled ZnO nanoparticles had good selectivity along with a sensitivity towards chloroform (CHCl_3_) and ethanol at a 5 ppm testing level, a value of concentration often found in patients suffering from cancer.

Another ZnO-based sensor has been reported for the detection of butanone (C_4_H_8_O), a VOC present in the breath of patients with gastric cancer [202]. In particular, a bicone-like ZnO structure was prepared through a microwave-assisted template free method. The structure showed outstanding performances in terms of selectivity, sensitivity, and detection limit (0.41 ppm). 

## 5. Conclusions: Challenges and Opportunities

Global warning, overpopulation crisis, the decreasing availability of water, food fraud and adulteration, the overspreading of non-communicative diseases, are just some of the challenges the world is currently facing. In the most recent period, also influenced by important changes caused by the COVID-19 pandemic, society has gained more consciousness about these issues and has started asking its policy makers for relevant responses. Thus, sustainable development has become a primary necessity, not just a desirable eventuality.

Scientists have been undoubtedly among the first suggesting key strategies for a green future. In the field of analytical chemistry, researchers have specifically highlighted the importance of accessing sustainable, innovative, fast, and accurate techniques and technologies for VOCs’ analysis alternatives to the traditional tools requiring expensive, long analysis, and that imply the disposal of large volumes of waste (e.g., solvents), such as mass spectrometry, adsorption/atomic emission spectroscopy or chromatography-based techniques (Table 3).

As described throughout this review, in recent years researchers have proposed novel sustainable sensors and biosensors for VOCs’ detection for highly relevant applications and for the well-being of society. The monitoring of the toxicity of different environments (e.g., houses and schools), the control of the freshness and quality of foods, especially in meat and fish products, and the diagnosing of different diseases such as diabetes or cancer, are just some of the potential uses of these new devices.

Remarkable results have been obtained, but still there are important barriers to overcome, including optimizing the selectivity, the stability, the efficiency and the detection limit of these sensors and biosensors. For example, there are inorganic gases, pathogens, or compounds such as proteins, that can interact with the devices and interfere with their specific sensing actions, affecting selectivity. Thus, these devices are required to differentiate target substances from non-targets, showing high specificity and reducing non-specific interactions. Additionally, most of the sensors and biosensors were developed without performing a deep analysis of the production and utilization costs, which can be higher than the production and utilization costs of classic analytical tools. Finally, it must be highlighted that little effort has been given to deeper explore and investigate the end life of these sensors and biosensors, which should be considered a crucial point in the development of this type of device.

In future development, these issues can be addressed by exploiting the most recent advances in the technologies related to the different components of the sensors and biosensors. For example, the latest results in biotechnology are opening to the possibility of designing highly selective biosensors by tuning the affinity of the biological receptors to selected VOCs thanks to gene editing techniques [203,204]. Additionally, progress in microfabrication can lead to a substantial decrease in production costs, to large scale fabrication of nominally identical structures, and to the possibility of integrating different sensors and biosensors [205]. Lastly, to fully attain the sustainable characteristics needed for sustainable development, a life cycle assessment (LCA), claimed to be the best framework for assessing the potential environmental impacts of products [206], must be also determined for all sensors and biosensors before being brought to the market.

Forthcoming optimized VOC sensors and biosensors can be thus employed for the monitoring of thousands of environments and microenvironments by performing analyses at low costs and with high efficiency. This can have a tremendous impact on society, for example, by monitoring the quality of air in sensitive places such as schools and hospitals, or by making possible the massive control of food quality in the food supply chain, breaking down the food waste. The integration of the newest sensors and biosensors with innovative technologies will also potentially expand and integrate their use. For example, in combination with the Internet of Things (IOT), the sensors and biosensors can allow the real time monitoring of VOCs present in different places with communication among devices. This may result in the performing of corrective actions such as the activation of a ventilation mechanism in response to the reaching of a toxic concentration of a VOC in an environment. Additionally, integration with blockchain technology can provide information for producers, distributors and consumers about the origin, production, and traceability of food products within one portable, inexpensive, and compact device.

## Figures and Tables

**Table 1:** Common VOCs and associated emission sources.

VOC	Typical Emission Sources
Propane	Gas grills; gas heaters
Butane	Gas grills; gas heaters; gas torches; end-life fridges, and freezers
Methyl chloride	Solvents; fire extinguishers
Formaldehyde	Plastic furniture items; fiberboards
Toluene	Paints; solvents
Acetone	Solvents; wallpaper and furniture polish
Isopropyl alcohol	Solvents; disinfecting solutions
Carbon Tetrachloride	Fire extinguishers; cleaning products
Carbon disulphide	Volcanic eruptions; marshes
Vinyl chloride	PVC pipes, wire, cable coatings, and textiles; burnt tobacco
Benzene	Fuels
Styrene	Polystyrene objects, rigid panels, and furnishings
Acetic acid	Cellulosic materials such as wood and paper
Isoprenoids	Plants

**Table 2:** Some of the most abundant VOCs normally found in indoor environments such as houses and schools.

VOCs	Maximum Concentration (µg m^−3^)		
	Houses According to Héroux et al. [97] *	Houses According to Yamazaki et al. [10] **	Primary School [11]
Toluene	436	530	117
Dichloromethane	1687	/	/
α-pinene	801	/	506
Limonene	329	/	/
Dichlorobenzene	287	4900	/
Tetrachloroethylene	179	/	/
Styrene	14	2000	369
Formaldehyde	/	100	/
Acetaldehyde	/	150	/
Cumene	46	/	/
Ethylbenzene	20	590	196
Hexane	39	/	/
Naphthalene	23	/	/
n-decane	203	/	/
Xylene	77	310	153

* Houses located in Quebec, Canada, ** Houses located in different cities in Japan.

**Table 3:** Advantages and disadvantages of classic tools and sustainable sensors and biosensors for the detection of VOCs.

	Advantages	Disadvantages
Classic methods (e.g., GC, HPLC, PTR, etc.)	High specificity; rapid separations; robust techniques	Matrix effects; high costs; higher maintenance; laborious sample preparation
Sustainable sensors and biosensors	Rapid response and recovery time; inexpensive; high sensitivity; small size; good precision; robustness	Temperature and humidity sensitive; high power consumption; short lifetime

## Data Availability

Not applicable.
